# Medical Gossip

**Published:** 1861-01

**Authors:** 


### Art. XIII.—MEDICAL GOSSIP.

The College of Physicians of London, which had been described as a corpse galvanized into activity by the fear of annihilation under the Medical Act, has given a fresh proof of its real vitality. It had already sought a cure by transfusion of new blood into its veins; and now, invigorated by the great accession of members and the large addition to its coffers of the precious 'circulating medium' obtained by the creation of a batch of new fellows, and the admission of a still larger number of licentiates, the Council have resolved to strengthen their position yet further by bringing into relationship with their corporation the great body of general practitioners. Their determination to create a new order of licentiates, who shall not be restricted from supplying medicines to their own patients was a stroke of the most politic ingenuity. It had not the merit of originality, since it had long been urged upon them by the *Lancet* and other journals; but nothing could tend more strongly than this proceeding to entrench the College in the strong affections and interests of the great mass of the profession of England. The Apothecaries Company in Blackfriars of course took fire instantly at a blow struck so vigorously against the flinty surface of their proper interest. They announced the determination to oppose, *vi et armis,* or yet worse, *lege et togâ,* the exercise of the proposed powers, which they declared to be vested solely in themselves by the Apothecaries Act of 1815, and by their revised charter. Of course, counsel's opinion was taken upon both sides; and perhaps also, as a matter of course, the question was rendered yet a little more complicated and dubious by the comparison of the adverse opinions so obtained from men equally eminent in the law. It may certainly be accepted as a by no means surprising result, that both parties found, in the opinions tendered, encouragement to proceed in the course of their already declared wishes, and try the question in a court of law. At first the College of Physicians resolved to adopt the timid course of asking for a decision of the courts before they proceeded to create their licentiates, but quite recently we learn that they have taken heart of grace, and are prepared to license practitioners in medicine, who may dispense the required remedies to their own patients, and of course, also, to accept the cost and responsibility of defending any action which may be set on foot to punish or prevent his exercise of those functions. This is probably at once the bolder and more prudent step.

Meantime, the College of Physicians of Edinburgh are actively watching over the interests of the thousand licentiates whom they admitted pell-mell in the year of grace, and have addressed a remonstrance to Mr. Secretary Herbert, who had expressed his intention of conceding a higher position in the estimation of the authorities of the Military Medical Department to graduates in medicine than to licentiates. Mr. Sidney Herbert is known to seek severe professional advice on such points, and his answer has indicated a clear appreciation of usances, which cannot be observed in a public department without the occasional infliction of injustice. After all, it must be remembered that the choice of an alma mater is not always voluntary; it is as often a question of money as of mind. A graduate of the oldest and highest universities is not necessarily a man of higher attainments than a licentiate of one of the corporations. And when once the standard of qualification is fixed, military medical officers may expect to be judged by the capacity which they show on entering the service and the zeal and ability with which they execute their labours, rather than by the source of their degree or the seat of their education.

The vexed question of medical titles remains still in a condition of chronic irritation. The relative proportions of right or courtesy involved in the bestowal of the cabalistic initials M.D. are the subject of constant controversy. In the case of the King's and Queen's College of Physicians of Ireland, Mr. Denny, the Irish Attorney-General, has given a formal opinion that the licentiates and fellows of that college are entitled to the degree and title of Doctors in Medicine, and to use the letters M.D. after their name. The charter of this college is exceptional in its wording; but the privilege being confirmed to the one college, there seems no great reason why it should not be extended to others more venerable and equally efficient.

The legal questions connected with the improper assumption of the title of Doctor have been tried more than once in the present term, and with uniform failure to procure conviction under the Medical Act. Not only has it been decided on the dictum of the Lord Chancellor that a practitioner being registered may call himself Doctor, without liability to any penalty, although he be neither a licentiate nor graduate of a medical college, but it has been affirmed by Mr. Selfe, the London magistrate, in a still more recent decision, that the fact of practising medicine, together with the simultaneous assumption of the title of Doctor by a person wholly unqualified, is not an offence under this Act, when this cannot be construed into a charge that the person, so acting, implied that he was registered. So awkward and double-edged a weapon was dearly purchased by the profession at the cost of forty thousand pounds.

The introductory lectures of the new session 1860-61, were delivered this year to a more numerous auditory than has for a long time graced the benches of the medical schools of the metropolis. The lecturer at St. Bartholomew's was Mr. Savory; at Guy's Hospital, Dr. Wilks; at King's College, Dr. Johnson; St. George's, Dr. Pitman; Charing Cross, Dr. Chowne; Middlesex, Dr. Stewart; University College, Mr. Erichsen; St. Mary's Hospital, Dr. Tyler Smith; Westminster Hospital, Mr. Power; Grosvenor-place School of Medicine, Mr. Lane. The Army Medical School at Chatham was opened by an address from Mr. Longmore, Professor of Military Surgery in that educational institute. The lecturers adopted for their themes subjects of the most various interest. Mr. Savory illustrated the text that genius means only transcendant power of taking trouble, first of all, by illustrations drawn from the life of the most illustrious philosophers, warriors, and physicians. Dr. Pitman introduced a variation of the same *thema*. Dr. Wilks attacked warmly the doctrine of specialities, and thought the English profession were more than ever open to the ridicule of Goldsmith, who said that in his time, in England they had "one doctor for the eyes; another for the toes; their sciatic doctors, and inoculating doctors; one doctor who is modestly content with securing them from bug-bites, and five hundred who prescribe for the bite of mad dogs." Mr. Grainger took his leave of a school in which he has long and ably taught, in an oration characterized by lofty and nobly inspired reflections upon the relations of life and matter. The address of Dr. Johnson bestowed upon the late Dr. Todd a well-merited apotheosis.

Mr. Hilton has, in his quality of Professor of Anatomy and Surgery to the College of Surgeons of England, delivered a series of lectures on "Pain and the Therapeutic Influence of Mechanical and Physiological Rest in Accidents and Surgical Diseases." Rest he regarded as the necessary antecedent of repair and growth. Borrowing his first illustration from cerebral disorders, Mr. Hilton recalled the frequency with which rest is a sufficing cure for mental disorders arising from overwork. He regarded the elastic capsules of the visceral organs, as a means of relieving them from the vascular turgescence which accompanies their temporary activity, and thus restoring them to physiological rest. The cerebro-spinal fluid was regarded similarly as a mechanical support to the internal parts of the brain, when they ceased have to be in the condition of physiological turgescence, and, supported by the cerebral circulation, as tending to bring back these internal parts of the brain to a state of comparative emptiness or quiescence after their state of activity.

Passing to practical and pathological illustrations of the value of rest, Mr. Hilton related numerous interesting instances of diseases of the spinal column, painful ulcers, and other surgical affections, in which the diagnosis or the cure have been due to the study of pain as a symptom, and rest as a therapeutic means. This course of lectures has been marked by great ingenuity, and philosophic and original research.

Of other lectures for the quarter, there have been the Lettsomian lectures on "Medicine," by Charles J. Hare, M.D., entitled "Practical Observations on some of the Points of Difficulty in the Investigation and Diagnosis of Tumours and Intumescence of the Abdomen."

The lectures at the London College of Physicians are to be delivered as follows, during the ensuing year. The Lumleian Lectures by Dr. Barker, Croonian Lectures by Dr. Guy, and the Gulstonian Lectures by Dr. Brown-Séquard. Short courses of lectures will also be given by Dr. Garrod and Dr. Lionel Beale.

The neglected state of the last resting-place of the illustrious Harvey at Hemel-Hempstead, has been the subject of much discussion since Dr. Tyler Smith drew professional attention to its indecorous disorder. The necessary repairs have, we believe, been executed, but a correspondence is in course between the

College of Physicians and the descendants of the great English physiologist, having for its object the removal of the remainn Harvey to Westminster Abbey.

The retirement of Ricord from the post which he has occupied at the Venereal Hospital of Paris for the last twenty-five years, has been attended with wide-spread demonstrations of respect and esteem for the eminent syphilographer. His retirement has raised anew on this side of the ehannel the question of the superannatioun of physicians and surgeons of hospitals as regulated in France, where physicians are expected to retire at sixty-five, and surgeons at sixty. The oft-expressed opinion is repeated that at such an age surgeons and physicians have done all that they can do efficiently for their hospital, and the hospital has done all that it can do for them. It is time that they should make way for other men, long and faithful servants of the poor, who are now too often withheld from posts to which they have a natural claim. The assistant-surgeons and physicians of the London Hospitals continue to complain loudly and justly of their anomalous position, which demands of them during a long series of years the most arduous labours, and considerable expenditure of time, while it imposes an often almost life-long inferiority of title and grade, together with a total denial of remuneration. This is a relic of the old days of oligarchy and nepotism, when the whole power was in the hands of a few in the hospitals, and there were many who were content to serve in bondage for "seven years and more," in the hope of rewards in the future. At King's College and St. Mary's Hospitals, both of which include in their managements some *esprits novateurs,* progress has been made towards remedying the evil.

The newly published list of the College of Surgeons of England shows that 249 members have been admitted fellows of examination, 16 more than last year. There are now 1267 fellows and 14,000 members, 769 licentiates in midwifery, and 100 licentiates in dental surgery.

The annual balance-sheet of the College has also just been published. The receipts amount to 22,307*l.*, of which 4756*l.* is derived from the sale of stock: the expenditure to 21,906*l.*, of which 7761*l.* is devoted to purchase of additional premises; while of the rest no less than 9215*l.* is devoted to "College department, including Council, Court of Examiners, Dental Board, &c.," and only 634*l.* to the "Library, including purchase and binding of books, salaries, &c." Truly these gentlemen are very careful that they be themselves well paid and well fed, and devote more to their own material support than to the intellectual alimentation of all their members.

The quarter has not been very fruitful in miscellaneous medical news.

The Social Science Association, which held its meeting at Glasgow early in the quarter, was fairly attended by medical men, especially in its sanitary section. Besides the local members of the profession, Sir Charles Hastings and Dr. Lankester read papers on Sanitary Science. The subject of provision for idiots and imbeciles was carefully discussed in a paper presented by Dr. Brodie, who estimates this class at 12,000 persons in England and Wales, for whom provision exists for only 600. The Glasgow faculty took occasion to protest against the present system of compelling medical practitioners in Scotland to furnish gratuitous certificates of death.

In journalism the *Akbare Jubabut* is the latest announced addition to our medical literature. This quaint periodical sheet is a monthly publication, intended to form a medium of communication between the native doctors in Government employ in India and native hakeems, with the view of spreading surgical and medical knowledge among the natives for the alleviation of their diseases. We heartily wish it success. The last report of Dr. Hobson's Missionary Hospital in China speaks of great success among a less promising population, and probably it might be well to consider the propriety of founding similar establishments in India. A new *Natural History Review* is announced in London, in the editing of which a part will be taken by Dr. Carpenter, Mr. Busk, Professor Huxley, and others.

We are glad to hear that the Army Medical Department is preparing, under the energetic rule of Dr. Gilson, and with the able help of Dr. Mapleton, Dr. Balfour, and Mr. Fitzgerald, to bring out a first printed annual report of the health, sickness, and death rate of the British army at home and abroad. Such returns will be highly interesting, as bringing the army henceforth under the critical observation of civil sanitarians, and furnishing reliable data for discussion.

The general state of health of the troops in China has been admirable, testifying at once to the excellence of the arrangements made by Sir John Little and Dr. Gilson for the services under their respective control, and to the importance of giving due weight to the counsels of the medical department in all that can affect the health of the army. This lesson was taught in the Crimea at a terrible cost. It would seem, at least, not to have been learnt in vain.

The long agitation of the militia surgeons has at length elicited an offer from the Secretary of State for War to give them commissions to the line, provided they pass the necessary examination. We fear that the proviso will render the promise nugatory in many cases where the boon is most needed. To call upon men long employed in active service to pass examinations instituted for young men fresh from college, is to apply a very unfitting test. This was long ago proved in the civil service, and is always kept in mind by the most ardent advocates of the competitive system, among whom we rank ourselves.

Among the civil appointments of the quarter we have to record those of—

Dr. Wollaston, as Physician to the South Staffordshire Hospital.

Dr. Letheby, as Analyst to the City of London.

Dr. Bence Jones, F.R.S., Secretary to the Royal Institution, in room of the Rev. John Barlow, F.R.S., resigned.

Mr. Daniel Oliver, F.L.S., Librarian at the Kew Gardens, to the Professorship of Botany at University College, vacant by the retirement of Dr. Lindley.

E. B. Gray, Esq., M.B.C.K., as Physician to the Radcliffe Infirmary, in room of Dr. Rolleston.

Dr. P. Chaplin, Physician to the English Hospital for Jews, at Jerusalem.

J. H. B. M'Leod, M.D., Professor of Surgery in Anderson's University, Glasgow.

Mr. Jonathan Hutchinson, joint Lecturer on Surgery at the London Hospital Medical College, in place of Mr. Critchett, resigned.

---

